# Autophagy protects against retinal cell death in mouse model of cytomegalovirus retinitis

**DOI:** 10.1186/s12886-019-1141-y

**Published:** 2019-07-10

**Authors:** Juan Mo, Sally S. Atherton, Liya Wang, Susu Liu

**Affiliations:** 1 Henan Eye Institute, Henan Eye Hospital, Henan Provincial People’s Hospital, People’s Hospital of Zhengzhou University, Zhengzhou, Henan Province 450003 People’s Republic of China; 2 Department of Cellular Biology and Anatomy, Medical College of Georgia, Augusta University, 1120 15th Street, Augusta, GA 30912 USA

**Keywords:** Murine cytomegalovirus, Retinitis, Autophagy, Retinal cell death

## Abstract

**Background:**

Extensive death of uninfected bystander neuronal cells is an important component of the pathogenesis of cytomegalovirus retinitis (CMV). Our previous results have shown that there is a functional relationship between autophagy and apoptosis during MCMV infection of retinal pigment epithelium (RPE). The purpose of this study was to determine whether autophagy plays a significant role in the death of retinal cells during MCMV retinitis.

**Methods:**

The retinas of adult BALB/c mice were infected with MCMV via supraciliary injection. Rapamycin, a mTOR inhibitor, was injected to MCMV-infected BALB/c mice intraperitoneally. Immunohistochemistry and western blot were performed to observe the spread pattern of virus in retinas and the levels of targeted proteins. Plaque assay was performed to determine the virus titer in different groups. Since *Atg5* is a key gene regulating autophagy, we bred *Atg5*^*flox/flox*^*; Nestin-Cre* mice to deeply elucidate the role of autophagy during MCMV retinitis. *Atg5*^*flox/flox*^*; Nestin-Cre* mice were genotyped and infected with MCMV. Immunohistochemistry was performed to observe the type of virus-infected cells and apoptosis in retinas during MCMV retinitis.

**Results:**

In MCMV mouse model, MCMV infection in outer nuclear layer (ONL) and inner nuclear layer (INL) in the retinas caused cleaved caspase 3 positive apoptosis, which is not co-localized with early antigen (EA) positive virus infected cells in rapamycin treated group. Rapamycin treatment increased the levels of LC3B-II by inhibiting mTOR and decreased the levels of cleaved caspase-3 during MCMV retinitis. However, virus propagation was not affected by rapamycin. In *Atg5*^*flox/flox*^*; Nestin-Cre* mice, RPE and glial cells were the main targets of viral infection, and number of EA positive retinal cells and TUNEL positive retinal cells was significantly increased compared to *Atg5*^*flox/+*^*; Nestin-Cre* mice though there was no difference of virus propagation between *Atg5*^*flox/flox*^*; Nestin-Cre* mice and *Atg5*^*flox/+*^*; Nestin-Cre* mice.

**Conclusions:**

Autophagy protects retinal cells from MCMV infection induced apoptosis through mTOR-mediated signaling pathway.

## Background

Cytomegalovirus (CMV) retinitis is a sight-threatening opportunistic infection in retina of those immunosuppressed patients who have the acquired immunodeficiency syndrome (AIDS), or organ transplantation, or malignant tumor and undergo chemotherapy [1–4]. Our previous work has shown the presence of necrosis during MCMV retinitis. Apoptosis has also been identified to be involved in the mechanisms of human CMV (HCMV) retinitis [5, 6] and mouse CMV (MCMV) retinitis [7, 8]. The previous studies in our laboratory have shown that most virus infected cells are not apoptotic [7]. However, the mechanism by which virus infected cells are kept alive is still not clear. Recent studies have highlighted the functional relationship between autophagy and apoptosis in a context-dependent fashion [9]. Our previous work also has demonstrated the cross talk between autophagy and apoptosis during MCMV infection of RPE cells, suggesting that autophagy plays a protective role in MCMV infection of RPE cells [10]. Those results raised our interest on studying whether autophagy is involved in protecting virus infected cells from cell death in in vivo model of MCMV retinitis.

Autophagy is a process which is essential to maintain the cellular homeostasis by transferring defective proteins, organelles or viral proteins into lytic vacuolar compartments for degradation [11–19]. More and more researches have shown that autophagy is a basal cellular process and is critical for the maintenance of retinal structure under physiological conditions [19–21]. Autophagy also is a potential therapeutic strategy for retinal neovascularization [22]. Recent studies have highlighted the association between autophagy and viral infection. By regulating cellular functions through the immune response, deposition of cellular compounds and/or cell death, autophagy has effect on viral infection [23]. And autophagy is also stimulated [24] or blocked [25] by viral infection. The previous work in our laboratory has demonstrated that autophagy is initiated by MCMV during the early time points of infection but is inhibited by MCMV during the late time points of infection. Autophagy inducer rapamycin decreases caspase 3-dependent apoptosis during MCMV infection of RPE [10]. However, it has not yet been determined whether autophagy is regulated by MCMV retinitis in vivo, and in turn, whether MCMV infection in retinas can be regulated by autophagy.

*Atg5*^*flox/flox*^*; Nestin-Cre* mice were used in this study to investigate the autophagic and apoptotic response of MCMV retinitis and the effect of autophagy on MCMV infection. *Atg5* encodes autophagy protein 5, required for formation of autophagosome in mammalian cells [26], deletion of which impairs the process of autophagy. In this mouse model, *Atg5* gene is specifically knocked out in the most retinal cells of developing retina [27] including neural cells, Müller glial cells, microglial and endothelial cells [28].

In this study, our results demonstrate that autophagy inducer rapamycin inhibits caspase 3-dependent apoptosis during MCMV retinitis. *Atg5* disruption in retinal cells decreases the ability for the clearance of viral proteins in the retina and also results in increased retinal cell death during MCMV retinitis. Loss of Atg5 function in the most retinal cells therefore increased the susceptibility of mice to MCMV retinal infection. Taken together, our in vivo results suggest that autophagy protects against MCMV pathogenesis by a mechanism that prevents retinal cells from MCMV infection-induced apoptosis by controlling the clearance of viral proteins.

## Methods

### Animals

Adult (6–8 weeks old) female BALB/c mice (Taconic, Germantown, NY) were randomly grouped to each experiment. Nestin-cre mice were bought from Jackson lab (Bar Harbor, ME, USA). *Atg5*^*flox/flox*^ mice were bought from Yoshinori Ohsumi lab. *Atg5*^*flox/+*^*; Nestin-Cre* were bred with *Atg5*^*flox/flox*^ to get *Atg5*^*flox/flox*^*; Nestin-Cre* mice (Primers for genotyping in Table 1). The nomenclature for *Atg5*^*flox/flox*^*; Nestin-Cre* mice is accordance with the previously publications [27, 29, 30]. Control female mice and experimental female mice (6–8 weeks old) in the same litter were grouped in all experiments. The abnormal mice beyond average range in terms of weight, body size and activity were excluded from the experiment. All mice were located in specific pathogen free animal room and were allowed unrestricted access to food and water. They were maintained on a 12-h light and 12-h dark cycle. All animal experiments were performed in accordance with the Guide For the Care and Use of Laboratory Animals (eighth edition), and all procedures in this study conformed to the ARVO Statement for the Use of Animals in Ophthalmic and Vision Research and were approved by the Institutional Animal Care and Use Committee of the Medical College of Georgia at Augusta University. Animals were anesthetized with 1.25 μl/g of a mixture of 42.9 mg/ml ketamine, 8.57 mg/ml xylazine and 1.43 mg/mL acepromazine in phosphate-buffered saline (PBS) before all experimental manipulations. Animals were euthanized using CO_2_ before enucleating eyes. Each group in each experiment had a minimum of 4 mice and experiments were repeated at least three times (Table 2).

**Table 1:** Sequences of primers used for genotyping mice

Gene	Primer sequence	Predicted band size (in bp)
Atg5 WT	Forward: 5′-GAATATGAAGGCACACCCCTGAAATG-3′; Reverse: 5′-GTACTGCATAATGGTTTAACTCTTGC-3′	350
Atg5 flox	Forward: 5′-ACAACGTCGAGCACAGCTGCGCAAGG-3′; Reverse: 5′-GTACTGCATAATGGTTTAACTCTTGC-3′	700
Atg5 deleted	Forward: 5′-CAGGGAATGGTGTCTCCCAC-3′; Reverse: 5′-GTACTGCATAATGGTTTAACTCTTGC-3′	320
Cre Internal CT	Forward: 5′-CTAGGCCACAGAATTGAAAGATCT-3′ Reverse: 5′-GTAGGTGGAAATTCTAGCATCATCC-3′	324
Cre	Forward: 5′-GCGGTCTGGCAGTAAAAACTATC-3′ Reverse: 5′-GTGAAACAGCATTGCTGTCACTT-3′	100

**Table 2:** Summary of mice (age and body weights)

Genotype	Number	Age	Mean body weight ± SEM (grams)
*Atg5* ^*flox/flox*^ *; Nestin-Cre* (homozygous, knockout)	4	6~8 weeks	13.10667±0.57897
*Atg5* ^*flox/+*^ *; Nestin-Cre* (heterozygous)	4	6~8 weeks	18.69±0.600722
*Atg5* ^*+/+*^ *; Nestin-Cre* (wild type)	4	6~8 weeks	20.224±0.29152

### Ocular inoculation

Each mouse was immunosuppressed with 2 mg sterile methylprednisolone acetate suspension by intramuscular injection every 3 days starting at day − 2 until the day when the eyes were enucleated. As shown in our previous paper, this treatment can deplete 93% of the CD4^+^ and CD8^+^ T cells as well as macrophages from MCMV-infected mice that were confirmed by flow cytometry of splenocytes [31]. The supraciliary route injection method was similar as previously described [32]. Briefly, by introducing the bevel of a sharp 30-gauge needle into the supraciliary space, a superficial transscleral entry wound was made parallel and just posterior to the limbus. The left eyes of mice were injected with 1 × 10^4^PFU of MCMV contained in a volume of 2 μl via the Virus (or medium) in a volume of 2 μl followed by 3 μl air using microinjection machine that was located in surgical room besides animal room. Right after injection, the injected eye was checked under dissecting microscope. The observation of chorioretinal detachment and the appearance of air in the supraciliary space suggest the success of injection. The mice with unsuccessfully injection were excluded from group. As for rapamycin treated group, rapamycin (1.25 mg/kg/day) was injected into mice every day via intraperitoneal injection. Mice were euthanized using CO_2_ at day 4, 7 and 10 p.i.. Injected eyes were enucleated and homogenized in serum-free tissue culture medium using a handheld tissue homogenizer (Biospec Products, Bartlesville, OK). Culturing homogenized eye with MEF cells was used to detect replicating virus. Mock (medium) injected eyes were grouped as controls and uninjected eyes of immunosuppressed mice were grouped as steroid controls. Eyes of mice in control group and experimental group were treated with eye ointment after surgery to prevent the infection and inflammation. The eyes were removed and prepared for immunohistochemistry or western blot analysis, as described below.

### Plaque assay

MEF cells were seeded into 24-well plates and incubated in Dulbecco’s modified Eagle’s medium (DMEM; Mediatech, Manassas, VA) containing 5% fetal bovine serum (FBS; Thermoscientific, Waltham, MA) at 37 °C in an atmosphere of 5% CO_2_ until the bottom of each well was covered by a monolayer of cells. Each eye that was infected with MCMV was collected at different time points and serially diluted. 100 μl of each dilution was added to the MEF monolayers. The cells were incubated at 37 °C for 1 h and gently shaken every 15 min during this absorption period. 1% Agarose solution was melted in a microwave and mixed with 2 × DMEM (Life Technologies, Grand Island, NY) with ratio 1:1. After incubation, the medium of each MCMV infected monolayer was discarded and 0.5 ml of agarose mixture (0.5% agarose in 1 × DMEM) was added to each well. After incubating for 5 days at 37 °C, 10% formaldehyde was added to each well for 15 min to fix the cells. The agarose overlay was removed. The fixed cells were stained with 0.13% crystal violet and plaques were counted under dissecting microscope.

### Virus propagation and virus titration

The original stock of MCMV (K181 strain) was a generous gift of Dr. Edward S. Mocarski (Emory University). The MCMV used in this study was prepared from the salivary glands of MCMV-infected BALB/c mice as described previously [33]. The titer of the virus stock was determined by plaque assay on MEF cells. Aliquots of stock virus were stored at − 70 °C, and a fresh aliquot was thawed and diluted to the appropriate concentration for each experiment.

### Western blot analysis

Proteins from retinas of MCMV-infected BALB/c mice, treated or not treated with rapamycin (Selleckchem, Houston, TX), or from retinas of MCMV-infected *Atg5*^*flox/flox*^*; Nestin-Cre* mice were extracted on ice by lysis buffer (Roche Diagnostics, Indianapolis, IN). Lysates were clarified at 13,000×g for 10 min at 4 °C and size-fractionated by 10% SDS-PAGE, and then electroblotted onto a polyvinylidene difluoride (PVDF) membrane (GE Healthcare, Pittsburgh, PA). The membrane was blocked with 5% nonfat dry milk for 1 h at room temperature, and then was incubated overnight at 4 °C with primary antibody (LC3B; Caspase-3; mammalian target of rapamycin (mTOR); phospho-mTOR; Cell Signaling, Danvers, MA). The next day, after three washes, the membrane was treated with HRP-conjugated secondary antibody 1 h at room temperature. The immune complex was visualized by a chemiluminescence detection system (Thermo Scientific, Waltham, MA) and was exposed to x-ray film. β-actin (Sigma-Aldrich, St. Louis, MO) was used for loading control among each sample. Each experiment was repeated three times.

### Immunohistochemistry

MCMV injected eyes, mock injected eyes and steroid control eyes were embedded in OCT compound (VWR Scientific, Radnor, PA). After snapping frozen in − 80 °C freezer, four frozen sections with 8 mm thickness on cryostat were collected to one slide. Frozen sections were fixed with 4% paraformaldehyde for 15 min.

The section was stained first with TUNEL (In Situ Cell Death Detection Kit, Fluorescein, Roche Diagnostics, Indianapolis, IN) according to the manufacturer’s instructions. After washing and blocking, biotinylated anti-MCMV early antigen (EA) (Sulfo-NHS-LC-Biotin; Pierce, Rockford, IL) [1, 34] was incubated in the section overnight at 4 °C. Texas Red-labeled avidin (Vector Laboratories, Burlingame, CA) was then used to bind to Biotin for 1 h at room temperature. The slides were then mounted with antifade medium containing DAPI (Vector Laboratories, Burlingame, CA) and examined microscopically.

Or the section was stained with fluorescein isothiocyanate (FITC; Sigma-Aldrich, St. Louis, MO)-conjugated EA and then stained with RPE65 antibody that was kindly provided by Dr. Michael Redmond (National Eye Institute, National Institutes of Health, Bethesda, MD), or stained with glial fibrillary acidic protein (GFAP) antibody (BD-Pharmingen, San Diego, CA).

### Hematoxylin and eosin (H and E) staining of ocular sections

The inoculated eyes were removed, snap frozen, and embedded in Tissue-Tek O.C.T. Compound (Sakura Finetek USA, Torrance, CA). Frozen sections were fixed in cold acetone for 5mins, and then stained with hematoxylin and eosin (H&E; Thermo Fisher Scientific Inc., Waltham, MA), washed, dehydrated in a graded alcohol series, mounted (Cytoseal; Richard Allan Scientific, Kalamazoo, MI), and allowed to dry overnight. Images were captured at 100-400× magnification with SPOT Advanced (Diagnostic Instruments, Sterling Heights, MI).

### Statistical analysis

Data for plaque assay, densitometry, number of immunopositive cells and TUNEL assay were expressed as means ± SEM (standard error of the mean) reflecting the results of independent experiments. In each case the data were reviewed to see how well they fit the assumptions of the tests. In most cases the comparisons were between multiple groups and the overall differences were analyzed by ANOVA using GraphPad Prism 5. The comparison between two groups was analyzed by two-tailed t-test using Microsoft Excel. A *p* value of *p* < 0.05 was considered significant. *p < 0.05, **p < 0.01, ***p < 0.001.

## Results

### Rapamycin protects against retinal cell death through induction of autophagy during MCMV retinitis

**Fig. 1:**
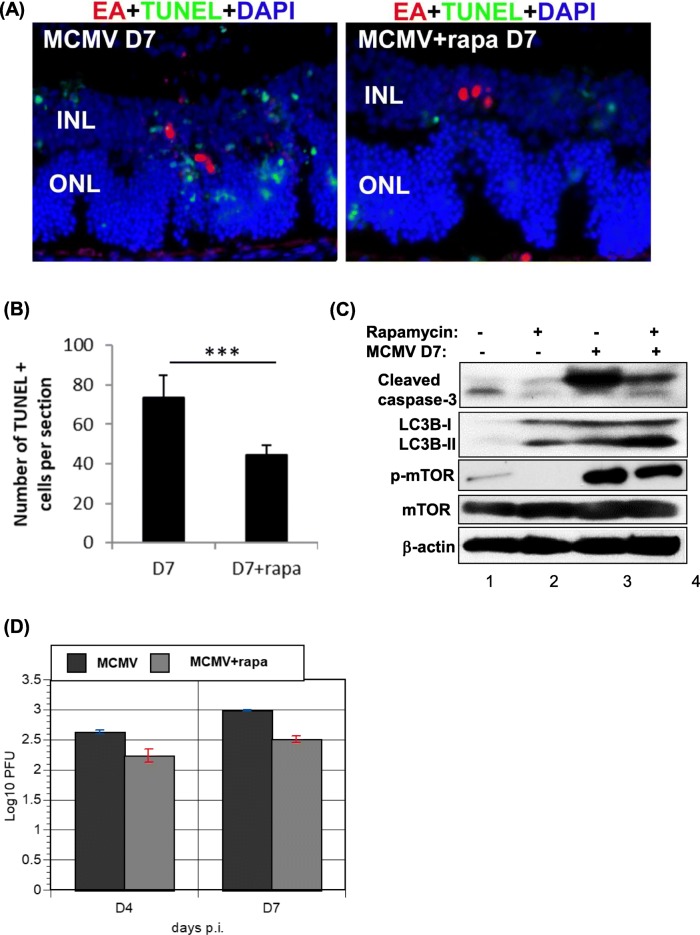
Effect of rapamycin treatment on mTOR, autophagy and apoptosis during MCMV retinitis. BALB/c Mice were immunosuppressed and injected via the supraciliary route with MCMV in a volume of 2 μl, and then injected with rapamcyin (rapa, 1.25 mg/kg/day) intraperitoneally every day. The inoculated eyes were collected at day 7 p.i. and prepared for fluorescent staining for MCMV early antigen (EA), TUNEL and DAPI (**a**). **b** The number of TUNEL-positive cells in rapamycin treated retinas was compared to that of non-treated retinas during MCMV retinitis. **c** Immunoblotting probed with antibody against LC3B, caspase 3, and phosphorylated mTOR (**c**), and virus titer (**d**) were performed respectively. The images in (**a**) are 400×. MCMV+rapa: MCMV infected and rapamycin treated mice

To investigate the role of autophagy in the pathogenesis of MCMV retinitis, BALB/c mice were immunosuppressed and MCMV was injected via supraciliary route to induce retinitis and rapamycin was injected into mice every day via intraperitoneal injection. Rapamycin positively regulates autophagy by inhibiting mTOR [35–38]. At day 7, we similarly observed EA positive cells in outer nuclear layer (ONL) and inner nuclear layer (INL) both in MCMV infected group and MCMV infected rapamycin treated group (Fig. 1a). The number of TUNEL-positive cells in rapamycin treated retinas was lower than that of non-treated retinas (Fig. 1b).

Autophagy [39, 40] has been approved to either reduce podocyte injury or inhibit cell death in several disease models; we therefore investigated whether autophagy also plays a protective role in our mouse model of MCMV retinitis. The western blot results showed that MCMV infection increased the levels of cleaved caspase 3 and the phosphorylation levels of mTOR at day 7 p.i. (Fig. 1c, compare lane 3 to lane 1). However, rapamycin treatment increased the levels of LC3B-II which is the marker for autophagy and decreased the phosphorylation levels of mTOR in MCMV infected mice compared to untreated mice at day 7 p.i. (Fig. 1c, compared lane 4 to lane 3), meaning rapamycin treatment increased autophagy by inhibiting mTOR pathway during MCMV retinitis. Meanwhile, rapamycin treatment decreased the levels of cleaved caspase 3 in MCMV infected mice compared to untreated mice at day 7 p.i. (Fig. 1c, compared lane 4 to lane 3). Those results are consistent with what we have found in MCMV infected RPE cells [10, 41]. However, virus titer in rapamycin treated MCMV infected mice was not statistically different from that in untreated MCMV infected mice (Fig. 1d). Taken together, these results suggest that the activation of mTOR pathway and the induction of caspase 3-dependent apoptosis are involved in the process of MCMV retinitis. Induction of autophagy by rapamycin through the inhibition of mTOR pathway reduces MCMV infection-induced caspase 3-dependent apoptosis.

### Autophagy is essential for the clearance of viral proteins from the retinas along with progression of MCMV retinitis

**Fig. 2:**
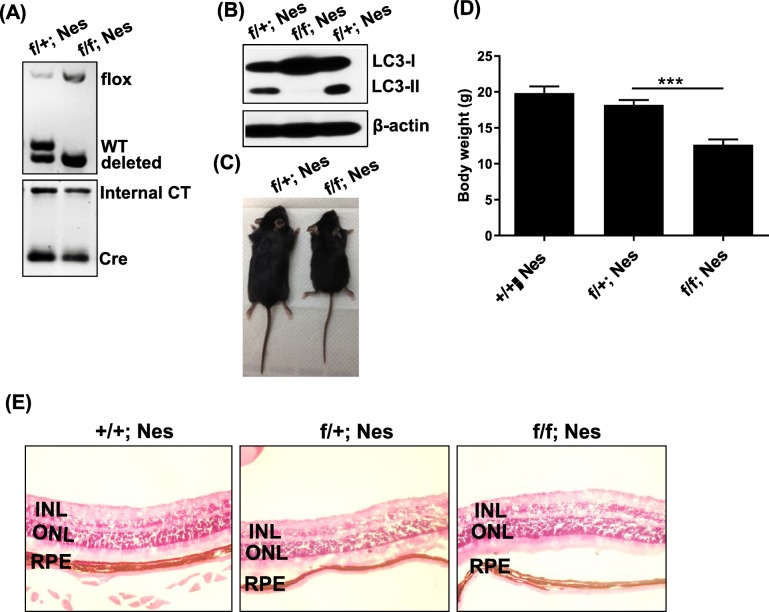
Characterization for Atg5 mice. **a** Ear DNA was extracted from Atg5 mice for genotyping. **b** Retinas were isolated from Atg5 mice. Expression levels of LC3B were monitored. **c** *Atg5*^*flox/flox*^*; Nestin-Cre* mouse was smaller than *Nestin-Cre; Atg5*^*flox/+*^ control mouse. **d** Body weight (gram) of Atg5 mice was measured and comparison was analyzed. The images in (**e**) are 100×. +/+; Nes: *Atg5*^*+/+*^*; Nestin-Cre*; f/+; Nes: *Atg5*^*flox/+*^*; Nestin-Cre*; f/f; Nes: *Atg5*^*flox/flox*^*; Nestin-Cre*

**Fig. 3:**
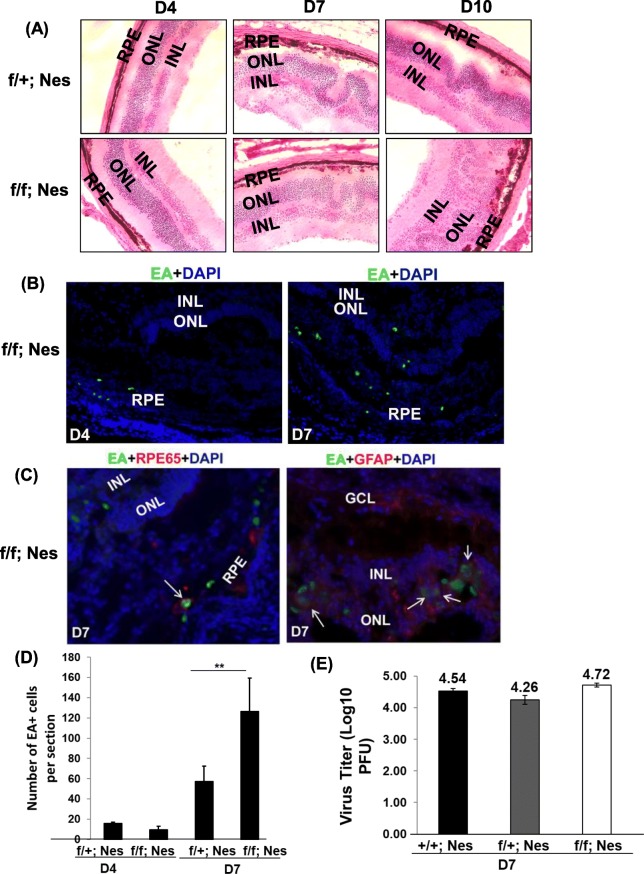
MCMV infection pattern in the retinas of *Atg5*^*flox/flox*^*; Nestin-Cre* mice during MCMV retinitis. Mice were immunosuppressed and injected via the supraciliary route with 1 × 10^4^ PFU of MCMV in a volume of 2 μl. The inoculated eyes were collected, and H&E was performed (**a**), and stained for MCMV early antigen (EA) and mounted with DAPI (**b**), or stained for MCMV early antigen (EA), RPE65 or GFAP, and mounted with DAPI (**c**). The images in (**a**) are 200×. The images in (**b**) and (**c**) are 40× and 200×, respectively. **c** Quantification of number of EA-positive cells in the retinas during MCMV retinitis. The number of EA-positive cells was counted in six sections from each mouse (4 mice in total). **d** Virus titer was measured in wildtype, heterozygous and homozygous mice for three repeats. RPE: retinal pigment epithelia; ONL: outer nuclear layer; INL: inner nuclear layer

To gain insight into the role of autophagy in general, and Atg5 in particular, in the protection from MCMV infection-induced apoptosis during MCMV retinitis, *Atg5*^*flox/flox*^*; Nestin-Cre* mice were bred according to the protocol [27]. The *Atg5* gene is knocked out in retinal cells of developing retina [27] including neural cells, Müller glial cells, microglial and endothelial cells [28]. Genotyping (Primers are shown in Table 1) results showed that *Atg5* gene was conditionally knocked out in *Atg5*^*flox/flox*^*; Nestin-Cre* mice (Fig. 2a). *Atg5*^*flox/+*^*; Nestin-Cre* mice were used as control. Western blot further confirmed that there were lower levels of LC3B-II in the retinas of Atg5 deficient mice compared to that of control mice (Fig. 2b). Body weight examination showed that *Atg5*^*flox/flox*^*; Nestin-Cre* mice were smaller and lighter than control mice (Fig. 2c and d). The retinas of *Atg5*^*flox/flox*^*; Nestin-Cre* mice were as normal as the control mice (Fig. 2e). Control mice and *Atg5*^*flox/flox*^*; Nestin-Cre* mice with 6~8 weeks old in the same litter were chosen for experiments (Table 2). The mice were immunosuppressed with steroid and injected with MCMV to induce retinitis. Then mice were sacrificed at day 4, 7 and 10 p.i.. The H&E showed that *Atg5*^*flox/flox*^*; Nestin-Cre* mice had more folding and more severe damage of retinas after MCMV infection compared to control mice (Fig. 3a). The samples were stained with MCMV early antigen (EA) and different markers for retinal cells. The immunostaining results showed that RPE layer was the first target of viral infection at day 4 p.i., and then virus spread from RPE to ONL and INL at day 7 p.i. (Fig. 3b). RPE65-positive RPE cells and GFAP-positive glial cells were the main targets of viral infection (Fig. 3c). Moreover, we observed infiltration between RPE and ONL in *Atg5*^*flox/flox*^*; Nestin-Cre* mice at day 7 p.i. (Fig. 3b and c), indicating that autophagy is associated with immune response, which contributes to the pathogenesis of MCMV retinitis.

At day 4 p.i., no difference in the number of MCMV EA-positive cells was observed in the retinas between *Atg5*^*flox/flox*^*; Nestin-Cre* mice and control mice after viral infection (Fig. 3d). At day 7 p.i., we observed the significant increase in the number of MCMV EA-positive cells in the retinas of *Atg5*^*flox/flox*^*; Nestin-Cre* mice compared to control mice after viral infection (Fig. 3d), though virus titer in *Atg5*^*flox/flox*^*; Nestin-Cre* mice was not statistically different from *Atg5*^*+/+*^*; Nestin-Cre* and *Atg5*^*flox/+*^*; Nestin-Cre* mice (Fig. 3e). Thus, these results support the idea that autophagy, Atg5 in particularly, is essential for the clearance of viral proteins from the retinas along with progression of MCMV retinitis.

### Autophagy deficiency results in more retinal cell death during MCMV retinitis

**Fig. 4:**
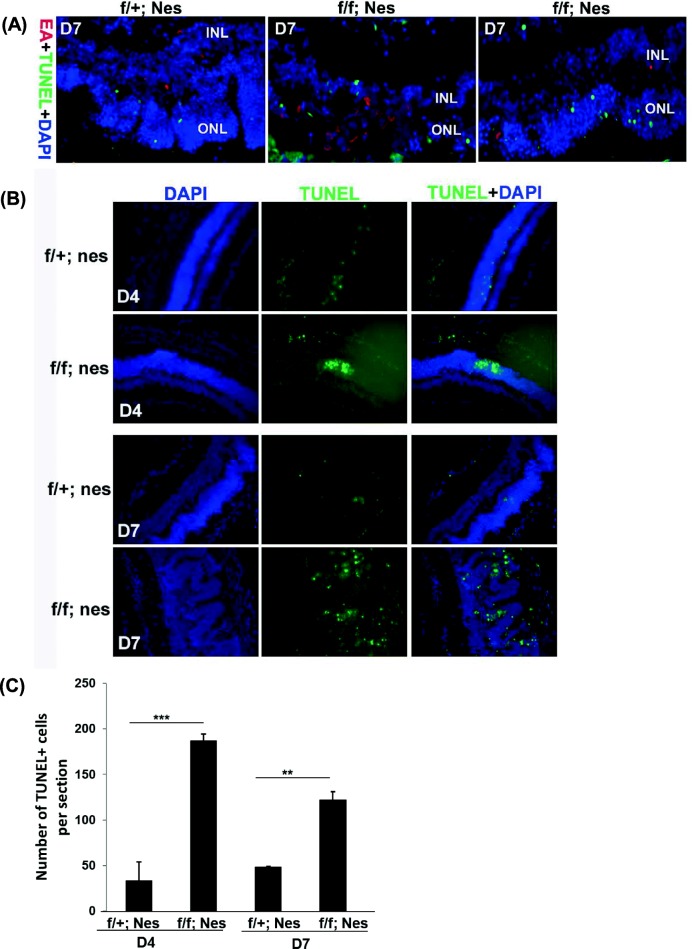
Apoptosis in the retinas of *Atg5*^*flox/flox*^*; Nestin-Cre* mice during MCMV retinitis. **a** Mice were immunosuppressed and injected via the supraciliary route with 1 × 10^4^ PFU of MCMV in a volume of 2 μl. The inoculated eyes were collected and stained for MCMV early antigen (EA), TUNEL and mounted with DAPI. The images in (**a**) are 400×. **b**-**c** Fluorescent staining for TUNEL and quantification of number of TUNEL-positive cells in the retinas during MCMV retinitis were performed at day 4 and day 7. The number of TUNEL-positive cells was counted in six sections from each mouse (4 mice in total)

To further investigate whether autophagy plays a protective role during MCMV retinitis, TUNEL assay was used to stain apoptotic cells in control mice and *Atg5*^*flox/flox*^*; Nestin-Cre* mice at day 4 and day 7 p.i.. We observed that EA positive MCMV infected retinal cells were not co-localized with TUNEL positive retinal cells in ONL and INL at day 7 p.i. (Fig. 4a, right panel). We also observed that a lot of retinal cells were lost in the area where there were many EA-positive cells in the retinas of *Atg5*^*flox/flox*^*; Nestin-Cre* mice (Fig. 4a, middle panel). Those results suggest that virus has strategy to protect the infected host cells from apoptosis; however, virus infection in retinal cells induces the apoptosis of bystander cells.

The fluorescent staining and quantification showed the significant increase of the number of TUNEL-positive cells in the retinas of *Atg5*^*flox/flox*^*; Nestin-Cre* mice versus control mice at day 4 and day 7 p.i. (Fig. 4b and c). The results that at day 4 p.i., no difference of the number of EA-positive cells (Fig. 3d) but more numbers of TUNEL-positive cells (Fig. 4c) in the retinas of *Atg5*^*flox/flox*^*; Nestin-Cre* mice versus control mice indicate that autophagy protects retinal cells from MCMV infection-induced apoptosis although autophagy could not clear viral proteins at day 4 p.i. during MCMV retinitis. Along with progression of retinitis, deficient autophagy resulted in increased number of EA-positive cells (Fig. 3d) and TUNEL-positive cells (Fig. 4c) in the retinas of *Atg5*^*flox/flox*^*; Nestin-Cre* mice versus control mice at day 7 p.i., suggesting that deficiency in autophagy impairs virus clearance and retinal cell survival along with progression of retinal damage.

Taken together, these results suggest that autophagy in general, and Atg5,in particular, protects against MCMV pathogenesis by a mechanism that prevents retinal cells from MCMV infection-induced apoptosis by controlling the clearance of viral proteins. Lack of Atg5-mediated autophagy renders mice more susceptible to MCMV infection-induced injury to retinas.

## Discussion

Previous studies have shown that autophagy is very important in the protection of plants [42] and *Drosophila* [43] against viral infection. Here, we demonstrate that autophagy plays a protective role during MCMV retinitis. Moreover, using *Atg5*^*flox/flox*^*; Nestin-Cre*, our results provide evidence for function of the ATG gene, *Atg5*, which is involved in the clearance of viral proteins and essential to protect mice against retinal MCMV infection. Thus, autophagy plays a conserved role in antiviral infection beyond its crucial function in homeostasis maintenance under physiological condition [44].

Recent genetic knockout or knockdown studies have addressed the cross talk between autophagy and viruses [45–47]. Autophagy may work as a potential antiviral mechanism that viral components are degraded via an autophagolysosomal pathway [48]. Or paradoxically, some viruses may utilize components of the autophagic machinery to benefit the viral replication [47–50]. However, it is still not clear whether autophagic components are utilized by virus to directly facilitate viral replication, or autophagic proteins have alternative function to regulate immune response, the indirect consequence of which changes viral replication. In our study, autophagy activation by rapamycin did not affect virus titer but did increase the clearance of viral proteins during MCMV infection in the retinas. It has been further shown that Atg5 is associated with clearance of viral proteins because knockout of *Atg5* gene in *Atg5*^*flox/flox*^*; Nestin-Cre* mice reduced clearance of viral proteins in the retinas, which suggests a unique mechanism by which autophagic genes protect host against viral infection, involving the clearance of viral proteins without directly controlling viral replication.

CMV has several strategies to inhibit apoptosis which together increase viral replication and presumably the pathogenesis of viral infection. Since recent reports suggest that autophagy may be an adaptation to avoid cell death [9, 51], we hypothesized that increased autophagy during viral infection might contribute to inhibition of apoptosis. We used two complimentary models-autophagy regulator (rapamycin) and autophagy knockout (*Atg5*^*flox/flox*^*; Nestin-Cre* mice) to mediate autophagy and then identified the impact of autophagy on apoptosis during MCMV retinitis. The results from two models prove our hypothesis that autophagy is the strategy taken by virus to escapes MCMV infection-induced apoptosis because autophagy induced by rapamcyin decreases apoptosis, and autophagy knockout increases apoptosis during MCMV retinitis. However, the low living rate of *Atg5*^*flox/flox*^*; Nestin-Cre* mice after birth slowed down the process of this study. We suggest using tamoxifen inducible *Atg5*^*flox/flox*^ *; Nestin-iCre/ERT2 *mice and *Bax−/−* mice in which Bax is involved in mitochondria-mediated apoptosis to further deeply investigate the functional crosstalk between autophagy and apoptosis, and the role of autophagy during MCMV retinitis in vivo.

## Conclusions

Taken together, the results from this study suggest that autophagy, and particularly *Atg5* gene, is involved in the clearance of viral proteins against host viral infection. Autophagy plays an important role to protect retinal cells against MCMV infection-induced apoptosis during MCMV retinitis. Therefore, autophagy might be the potential therapeutic target for MCMV retinitis treatment.

## Data Availability

The data and materials used in current study are available from the corresponding author on reasonable request.

## Abbreviations

AIDS: Acquired Immunodeficiency Syndrome
CMV: Cytomegalovirus Retinitis
H&E: Hematoxylin and Eosin
INL: Inner Nuclear Layer
MEF: Mouse Embryonic Fibroblast
ONL: Outer Nuclear Layer
PBS: Phosphate-Buffered Saline
RPE: Retinal Pigment Epithelium
SEM: Standard Error of the Mean
